# Respiratory Monitoring at Bedside in COVID-19 Patients

**DOI:** 10.3390/jcm10214943

**Published:** 2021-10-26

**Authors:** Davide Giustivi, Francesco Bottazzini, Mirko Belliato

**Affiliations:** 1 A&E Department ASST Provincia di Lodi, 26900 Lodi, Italy; 2 Department of Anesthesia, Critical Care and Emergency, Fondazione IRCCS Ca’ Granda Ospedale Maggiore Policlinico, 20122 Milan, Italy; francesco.bottazzini@gmail.com; 3 U.O.C. Anestesia e Rianimazione 2 Cardiopolmonare, Fondazione IRCCS Policlinico San Matteo, 27100 Pavia, Italy; m.belliato@gmail.com

**Keywords:** COVID-19, work of breathing, PSILI, respiratory effort, VILI, ultrasound, ARDS, noninvasive ventilation

## Abstract

The SARS-CoV-2 (COVID-19) pandemic has forced some reflections to be had surrounding the ventilatory support to be applied to certain types of patients. The model of two phenotypes, set out by Professor Gattinoni and colleagues, suggests that adequate monitoring of respiratory effort may play a key role in the treatment of respiratory failure due to COVID-19. An insufficient control of the patient’s respiratory efforts could lead to an aggravation of lung damage, mainly due to the possibility of generating Patient Self-Inflicted Lung Injury (PSILI) with a consequent aggravation of the pathological picture. Nevertheless, effectively monitoring the patient’s respiratory work, especially in nonintensive settings, is not easy. This article briefly describes some methods that allow the assessment of respiratory effort, such as the use of ultrasound and respiratory tests, which can be performed in nonintensive settings.

## 1. Introduction

Since the 31 December 2019, when the Chinese authorities informed the WHO of the presence of a cluster of patients with pneumonia of unknown origins [1], it soon became clear that correct respiratory support for COVID-19 patients played a fundamental role in their treatment [2]. After starting to stratify patients into different phenotypes depending on the severity of their clinical and radiological picture [3], it was possible to correlate the patient’s clinical phenotype to disease progression [4], establishing a direct relationship between progression of the clinical phenotype which evolves towards increasingly severe pictures of respiratory failure, and the course of the disease which leads to its inflammatory phase. Therefore, the modalities of respiratory assistance for the COVID-19 patient have also changed [5], and, alongside the need to ensure adequate oxygenation parameters for the patient, more and more attention has been paid to the control of the respiratory drive, even during the phases of noninvasive ventilation (NIV) [6].

According to this point of intervention, it appears to be clear how the proper monitoring of the patient’s respiratory effort (Work of breathing, WOB), plays a decisive part in assisting this type of patient, in order to substantially minimize the risk of Patient Self- Inflicted Lung Injury (PSILI) [7], which appears to be closely correlated with the patient’s excessive respiratory work [8]. The WOB can be represented with this elegant equation: WOB=∫P×V, in which P is meant as the sum of all the concurrent pressures (muscle pressure, transpulmonary pressure, airway pressure, etc.).

A recent computational study has shown how, in a model correlated to COVID-19 patients, the levels of transpulmonary and pleural pressure swing, driving pressure, and mechanical power typically correlated with VILI in mechanical ventilation can be achieved in spontaneously breathing patients with intense respiratory activity [9].

Therefore, different modalities of respiratory effort monitoring are illustrated, which can be used in different clinical scenarios.

## 2. Clinical Evaluation

Clinical parameters: respiratory rate, saturation values, abdominal respiration.

**Figure 1:**
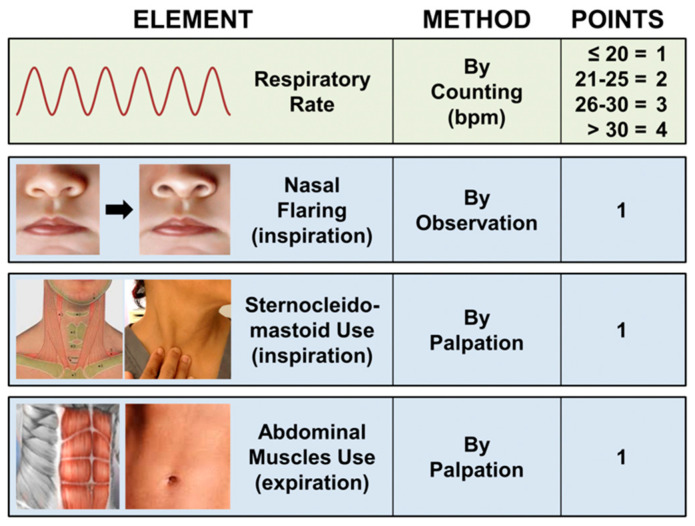
The WOB scale (courtesy of Gazmuri RJ).

- WOB scale [10]: developed specifically for COVID-19 patients and involves the observation of four different items (Figure 1), a score greater than 4 indicates a significant respiratory effort.

## 3. Pressure Assessment

Esophageal pressure swing (ΔP_ES_): the insertion of a balloon catheter into the esophagus allows the measurement of esophageal pressure (P_ES_), which is considered the most effective surrogate for pleural pressure (P_PL_) [11]. It is possible to observe both the absolute value at the end of inspiration or at the end of expiration, and the oscillations during the breaths (ΔP_ES_). It can be performed both in patients with fully controlled ventilation and in patients with spontaneous breathing [12]. In case of patients on controlled mechanical ventilation, the measurement of the esophageal pressure is used as a factor for the evaluation of the transpulmonary pressure (P_L_, obtained from the formula: P_L_ = P_AW_ − P_PL_), an effective indicator of the dynamic stress to which the lungs are subjected [13]. In patients with spontaneous breathing, the esophageal pressure, or rather the oscillations observed during breathing, are a good indexes of the work exerted by the respiratory muscles [14], and therefore are an effective indicator of respiratory effort [15], which, if it becomes excessive, leads to a progressive worsening of lung injury [7].

Central Venous Pressure swing (ΔCVP): the use of CVP oscillations as a substitute for pleural pressure (P_PL_) has been shown to be effective [16]. Furthermore, the use as a factor for the calculation of transpulmonary pressure, even though the population studied was of pediatric patients, proved possible [17], paving the way for new interpretations and use of this value [18].

Airway occlusion test: in patients with spontaneous breathing and subjected to mechanical ventilation, it is possible to perform forced closures of the respiratory circuit, which, depending on whether they occur at the end of inspiration or at the end of expiration, provides useful information on respiratory effort and energies applied to the lungs. An occlusion at the end of inspiration lasting > 3 s allows us to evaluate the plateau pressure (P_PLAT_), which as noted by Bellani et al. [19], when observing the pressure curve on the ventilator monitor, represents an index that can be used in patients with spontaneous breathing modes. The plateau pressure measurement (P_PLAT_) allows us to calculate two useful indexes of the stress of the lungs: the driving pressure (ΔP), even if the real usability in spontaneous breathing patients is the subject of discussion [20], and the Pressure Muscle Index (PMI), a comparable index with the advantage of a greater usability for detecting the pressure exerted by the respiratory muscles (P_MUSC_) [21]. The current reference standards for the quantification of the respiratory effort in the spontaneously breathing patient are described in the ATS/ERS statement of 2002 [22].

Instead, by performing an occlusion at the end of expiration [23], it is possible to evaluate the pressure generated in the first 100 ms of a spontaneous inspiratory effort of the patient (P_.01_), which is a direct indicator of the patient’s central respiratory drive [24]. The measurement of the P_.01_ value is useful both to evaluate an excessive assistance of the ventilator [25], and as an indicator of intense respiratory effort [26].

Observing the complete swing of the pressure wave, it is possible to measure the occlusion pressure (ΔP_OCC_), a new and promising detector of elevated transpulmonary driving pressure and pressure of respiratory muscles [27].

All the equations and reference values are shown in the table below (Table 1):

## 4. Volumetric Assessment

Vt: a careful monitoring of the tidal volume (Vt) is essential in the patient undergoing mechanical ventilation. As is well recognized, ventilation that produces large tidal volumes harms patients with lung lesions (e.g., ARDS) [28]. This is mainly due to two factors: mechanical rupture of the lung parenchyma with large barotrauma (pneumothorax, pneumomediastinum, subcutaneous emphysema) and pulmonary edema caused by lung over-distension (volutrauma) [29]. Both barotrauma and volutrauma belong to the category of VILI (Ventilator Induced Lung Injury).

Obviously, in mechanically ventilated patients with paralysis of the respiratory muscles, the energy applied for generation of the tidal volume is entirely due of the ventilator. While in the spontaneously breathing patient, it is shared between the ventilator and the patient’s respiratory effort, and the sum of these two pressures can contribute to the generation of dangerously high volumes [30] which can lead to a worsening of the patient’s respiratory ability and an increased risk of VILI [31]. In fact, in patients undergoing NIPPV it was noted that treatment failure is significantly correlated with the finding of large tidal volumes (i.e., >9.5 mL/Kg of PBW), particularly interesting is the fact that the author uses the expired tidal volume (VT_E_) as a parameter for measuring tidal volume, which it judges to be more reliable in patients undergoing noninvasive ventilation [32].

Furthermore, the evaluation of the Vt offers the possibility of evaluating the static lung compliance (C_STAT_ = $\frac{\mathrm{VT}}{\Delta P}$), a simple but useful indicator of the amount of the lungs participating in ventilation [33].

## 5. Ultrasound Evaluation

Thickening Fraction Index (TFi): by placing a linear probe in the apposition area (ZA) at the midaxillary line, the area in which the abdominal contents reach the rib cage, it is possible to view the diaphragm as a non-echogenic layer between two hyperechoic edges (peritoneum and pleura). At this point, with the M-mode or the 2D mode, it is possible to observe and evaluate diaphragm thickening during respiratory activity, both in inspiration (TEI) and in expiration (TEE), and the TFi can be easily calculated using the following formula: TFi = TEI − TEE/TEE [34].

Due to its linear correlation with lung volumes [35], the thickening fraction is widely used for the evaluation of diaphragmatic dysfunctions [36]. In the ICU it could be a tool for predicting successful weaning from invasive mechanical ventilation [37], and thanks to its close correlation with the esophageal time–pressure curve (PTP_ES_), and with the diaphragmatic time–pressure curve (PTP_di_) [38,39], the TFi may be used to monitor the muscular effort exerted by the diaphragm. Schepens et al. [40] suggested that a TFi > 0.30 should be considered a value that exposes the diaphragm to excessive stress with the risk of muscle trauma.

Diaphragm excursion (DE): by placing a phased array probe in the subcostal sagittal scan between the midclavicular line and the anterior axillary line and using the M-mode, it is possible to observe the movement of the diaphragm (DE) [41]. Additionally, this can be used in ICU for assessment of mechanical ventilation weaning [42]. As with TFi, DE also expresses a close correlation with lung volumes [43], and due to its speed and ease of execution it can be easily used outside the ICU for simple monitoring of diaphragmatic movement.

Even if the upper cutoff is not clearly expressed, it is possible to speculate, on the basis of normal values during a deep breathing [44], that ED values > 40 mm in women and >50 mm in men may be indicative of intense diaphragmatic activity.

Caval Index (CI): the measurement of CI is an attractive index, due to its close correlation with the PVC values [45], although the generation of strong negative intrathoracic pressures may be responsible for the collapse of the inferior vena cava (with an increase in CI values > 0.50) [46]. Particularly, in spontaneous breathing patients it is not correlated with vascular volume and it should be interpreted with caution, pending more certain data and differentiated from a circulating vascular volume problem.

**Figure 2:**
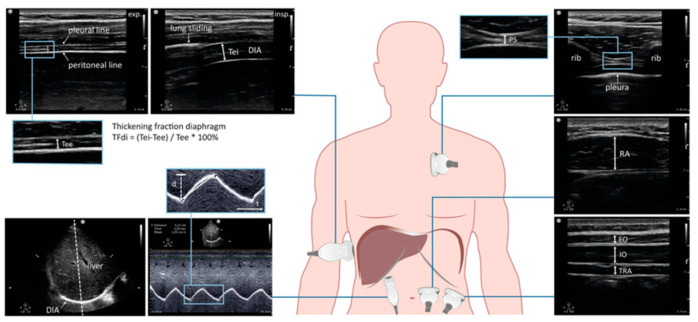
Clinical applications of respiratory muscle ultrasound (courtesy of Prof. Huiks Leo).

Accessory respiratory muscles: the decisive activation of the intercostal inspiratory muscles [47] and abdominal wall expiratory muscles [48], in case of intense respiratory work, can be described by ultrasonography [49] (Figure 2). It is also possible to measure the TFi value, but although interesting, more detailed data are needed.

## 6. Discussion

In a frame of a register of 1018 patients, coordinated by the IRCCS Ca’ Granda Policlinico Hospital of Milan and promoted by the Mario Negri pharmacological research institute [50], the percentage of patients who underwent oxygen therapy was equal to 69.6%, and 20.5% received treatment in the form of noninvasive ventilation. If the overall risk of in-hospital death was low (OR 0.84, 0.57–1.25), in patients undergoing NIV the risk appears significant (OR 4.31, 2.69–6.89). It is worth noting that a high respiratory rate at admission has a significant correlation with the risk of death (*p* value ≤ 0.001, OR = 1.13, 95% CI) [51]. Moreover, during the prospective one-day observational, WARd-COVID [52] described the data of 909 patients who underwent NIV out of intensive care, and found that 778 patients (85%) were treated with CPAP (of them 68% used a helmet as an interface). In this study, 300 patients who received the NIV approach had failed treatment (37.6%), with a mortality rate of 25%, and in the subset of patients who had failed treatment, the presence of dyspnea plus the use of accessory respiratory muscles were statistically significant (*p* < 0.001 for both). Based on these data and on the low levels of PaCO2 described (in 53.9% of the WARd-COVID patients), the authors share a reflection that the key point of assistance to the patient is the constant respiratory monitoring, which could offer a reduction of risks associated with tracheal intubation and the possibility of exposing the patient to PSILI.

In this review, we attempted to summarize the principally available tools for daily clinical practice of evaluating patients’ respiratory efforts, which we consider the most important diagnostic tool, both for patients under mechanical ventilation (where proper protective ventilation can be decisive) [15], and in spontaneously breathing patients [53]. In the latter, it is only possible to make an attempt to modulate the drive with the application of supplemental oxygen alone or with positive respiratory pressures (IPAP, EPAP; CPAP) at various levels and in different ways [54], and with the control of the drive with sedative drugs. Daily careful monitoring should be implemented to assess whether the patient will be responsive to treatment or whether they need an upgrade in respiratory support [55]. Furthermore, in accordance with the theory proposed by Luciano Gattinoni and colleagues [56] an effective control of respiratory drive could avoid the transition from a lung with low elastance and high compliance (phenotype L) to one with a high elastance and low compliance (H phenotype), both in the spontaneously breathing patient (P-SILI vortex) and in patients receiving mechanical ventilation (the VILI vortex).

## Figures and Tables

**Table 1:** Indexes and parameters with relative equation and normal values.

Index/Parameter	Equation	Normal Value
Esophageal pressure swing (ΔP_ES_)		3–8 cm H_2_O
Transpulmonary pressure (P_L_)	P_AW_ − P_ES_	<20 cm H_2_O
Central Venous Pressure swing (ΔCVP)		uncertain
Plateau pressure (P_PLAT_)		<30 cm H_2_O
Driving pressure (ΔP)	P_PLAT_ − PEEP	<15 cm H_2_O
Pressure Muscle Index (PMI)	P_PLAT_ − (PEEP + PS)	<6 cm H_2_O
P._01_		1.5–3.5 cm H_2_O
Occlusion pressure (P_OCC_)		Not defined

Abbreviation: P_AW_: Airway pressure, PEEP: positive end-expiration pressure, PS: pressure support.

## Data Availability

Not applicable.
